# Cluster randomised controlled feasibility study of HENRY: a community-based intervention aimed at reducing obesity rates in preschool children

**DOI:** 10.1186/s40814-018-0309-1

**Published:** 2018-06-21

**Authors:** Maria Bryant, Wendy Burton, Michelle Collinson, Suzanne Hartley, Sandy Tubeuf, Kim Roberts, Annemijn E. C. Sondaal, Amanda J. Farrin

**Affiliations:** 1 Clinical Trials Research Unit, Leeds Institute of Clinical Trials Research, University of Leeds, Leeds, LS2 9JT UK; 2 Academic Unit of Health Economics, Leeds Institute of Health Sciences, University of Leeds, Leeds, LS2 9JT UK; 3 HENRY, 8 Elm Place, Old Witney Rd, Oxfordshire, OX29 4BD UK; 4 The National Centre for Social Research (NatCen), London, EC1V 0AX UK

**Keywords:** Obesity, Trial, Feasibility, Childhood, Prevention, Community

## Abstract

**Background:**

In the UK and beyond, public funding is used to commission interventions delivered in public health early years settings aimed at improving health and well-being and reducing inequalities in order to promote school readiness. This is a key setting for obesity prevention programmes, which are often commissioned despite the limited evidence base. The HENRY (Health, Exercise, Nutrition for the Really Young) programme is an 8-week programme delivered to parents of preschool children, designed to support families to optimise healthy weight behaviours. Early evidence suggests that it may be effective, but a robust evaluation using a randomised controlled design has not been conducted. This study begins this process by evaluating the feasibility of conducting a multi-centre definitive trial to evaluate the effectiveness and cost-effectiveness of HENRY to prevent obesity in the early years.

**Methods:**

This is a multi-centre, open labelled, two group, prospective, cluster randomised, controlled, feasibility study aiming to recruit 120 parents from 12 children’s centres, based in two local authority areas. Within each of the two local authorities, three centres will be randomised to HENRY and three will be randomised to a control arm of standard care (usual provision of services within children’s centres). We will explore HENRY commissioning, provision and delivery and assess the feasibility of local authority, centre and parent recruitment, the processes and time required to train and certify staff to deliver the intervention, the potential sources (and associated risk) of contamination and the feasibility of the trial procedures. Research includes a process evaluation, feasibility of cost-effectiveness evaluation, with progression to the definitive trial judged against pre-defined criteria.

**Discussion:**

This feasibility study will support the decision to proceed to, and the design of, a future definitive trial, providing an evidence base of an approach to prevent childhood obesity, which has been deemed attractive to all stakeholders, including parents. Given the widespread adoption of the intervention, this has the potential to impact on public health in the UK and beyond.

**Trial registration:**

ClinicalTrials.gov Identifier NCT03333733 registered 6th November 2017

Protocol date: 25th October 2017

Protocol version: 4.0

## Background

Tackling obesity is a key global public health priority, with international, national and local strategies aimed at reversing the trend of rising rates of childhood obesity [1–3]. While treating obesity remains a priority, there are persuasive arguments for prevention during childhood. Establishing healthy behaviours in early childhood is essential to ensure optimal growth and development [4], and negative lifestyle behaviours that are developed early can persevere and are associated with chronic diseases in adulthood such as cardiovascular disease and type 2 diabetes [5]. Furthermore, once established, obesity is difficult to reverse [6], strengthening the case for prevention [7]. Interventions delivered in the first few years of life are therefore essential to impact the health of our children now and in the future [8].

Interventions aimed at preventing or treating obesity report inconsistent results [6, 9–13], though there is agreement that multi-component interventions especially those engaging parents have the greatest impact [6, 14]. Systematic review evidence has found that some efforts to prevent obesity have had disappointing results due in part, to inclusion of interventions which are not underpinned by a robust evidence-based and/or not rooted in behaviour change theory [6, 11–13, 15–17]. Intervention strategies are not always tailored to the most important and modifiable behaviours, and often, they are inadequate to change family, environmental and extrinsic factors in combination with health education strategies aimed at personal behaviours. Further, reviews indicate that there is a lack of careful pre-testing and formative evaluation procedures before larger-scale implementation and lack of involvement of stakeholders in the development. Interventions with greater involvement by parents/carers appear to offer the most promise in preschool children. The proposed feasibility study involves an independent evaluation of an existing preschool obesity prevention intervention, ‘HENRY’ (Health, Exercise, Nutrition for the Really Young). The HENRY organisation developed the HENRY programme in 2007 with the Department of Health and Department for Education support in the UK. It is an 8-week group-delivered programme provided to parents of preschool children and is currently commissioned and delivered across the UK by ~ 35 local authorities (approximately 10% of authorities) providing more than 190 programmes each year. Over 10,000 families have already participated. It is delivered in the community, predominantly by children’s centre staff [18]. HENRY uses a responsive approach to improve children’s centres environments and promote parenting skills aimed at enhancing family lifestyles. These intervention targets are consistent with literature in childhood obesity [7, 12, 13]. Preliminary data indicates that HENRY may be effective at reducing childhood obesity and improving family health [18], and research is underway evaluating engagement strategies with families [19]. However, research to date has not compared outcomes with those parents who have not attended HENRY; thus, further robust evaluation of effectiveness and cost-effectiveness using a RCT design is needed to establish its effectiveness.

Given the uncertainties associated with recruiting local authorities, service providers and children’s centres to a trial, it is necessary to conduct research in advance of a definitive multi-centre trial. The process of recruiting parents and conducting research in this setting also requires consideration, as models of commissioning and delivering HENRY are complex and vary by location. In some instances, HENRY is commissioned by local authorities and delivered by existing centre staff. In others, third sector or health providers may commission HENRY directly or locally employed HENRY staff may deliver programmes. This study will assess the feasibility of the planned recruitment rate at the local authority, children’s centre and parent level and ensure competence of programme delivery, in addition to providing vital information for calculation of the sample size for the future trial. There will also be a focus on identifying potential sources and risks of contamination and assessing the level of risk associated with each contamination source. A future trial will be used to offer commissioners an evidence-based intervention including a cost-effectiveness analysis, tested in children’s centres in the UK, and to provide much needed evidence on the effectiveness of early obesity prevention in public health settings.

## Methods/design

### Aim

This feasibility study aims to determine the feasibility of undertaking a definitive trial to evaluate the clinical and cost-effectiveness of the HENRY programme in preventing childhood obesity.

### Objectives

Primary objectives are the following:

1. To determine whether it is feasible to recruit local authorities/service providers that are willing to nominate children’s centres to be involved in the research (allowing randomisation in place of the usual selection of centres in most need).
2. To assess the time required to train and certify staff to competently deliver HENRY programmes, in order to propose a clear process and timeline for a definitive trial.
3. To determine whether it is possible to recruit parents to the study, who are enrolled to attend a HENRY programme, and the practicality of recruiting parents to the study from control centres, where the pathway to recruitment is less defined compared to the HENRY centres.
4. To explore HENRY commissioning, provision and delivery via postal screening questionnaires and qualitative data collection in areas currently delivering HENRY.
5. To explore potential sources and risks of contamination, including the degree to which parents use multiple centres, the level of contamination resulting from social networks (control and HENRY parents sharing knowledge) and the possibility of HENRY-trained facilitators sharing knowledge with control centres.

Secondary objectives are the following:

1. To examine the acceptability and completeness of the proposed methods of data collection to ensure they are feasible for a definitive trial.
2. To gather data to allow estimation of the sample size requirements for the definitive trial.
3. To assess the acceptability of the design for parents, commissioners and centre staff, particularly related to withholding HENRY training in control centres until the end of the study.
4. To determine the practicalities of delivering the required number of HENRY programmes within the trial period in regard to programme implementation.

### Progression rules for definitive trial

Progression will be judged via a traffic light system whereby ‘Green’ indicates it is feasible to proceed to a definitive trial with the current trial design and procedures, ‘Amber’ indicates modifications are required to the trial design and/or procedures before proceeding to a definitive trial, and ‘Red’ indicates it is not feasible to proceed to a definitive trial.

1. Recruitment of local authorities (and their service providers (if applicable)): Green = two local authorities within 12 months (beginning from receipt of ethical approval); Red = less than two local authorities within 12 months.
2. Randomisation of children’s centres: Green = at least 12 centres randomised within 12 months (beginning from receipt of ethical approval); Amber= 8–12 centres randomised within 12 months; Red = less than 8 randomised within 12 months.
3. Recruitment of parents: Green = an average of at least 4 parents registered per programme (or control group equivalent); Amber = 3 parents registered per programme; Red = less than 3 parents registered per programme.

Progression criteria have been approved by the steering committee. If any of the criteria are not met, future assessment of the effectiveness of the HENRY programme will be negotiated to decide if a definitive trial is feasible. If a definitive trial is supported, further feasibility testing may be needed prior to the conduct of a definitive trial should negotiations result in changes to the design based on failure to meet progression criteria. Our method for determining progression criteria are based on recommendations proposed by Avery et al. [20] in which these pre-specified criteria have been agreed with funders to determine progression to the definitive trial. Objectives related to staff training, collection of data and estimating sample size are included to support the design of a definitive trial, but do not have assigned progression criteria cutoffs. While important, they do not provide an indication of whether or not a future trial should proceed.

### Design

**Fig. 1:**
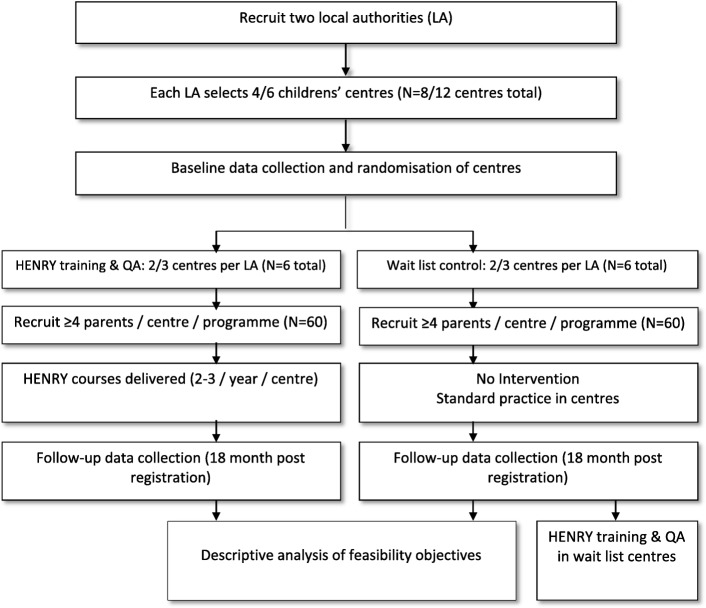
Flow chart of a cluster randomised controlled feasibility study evaluating feasibility/acceptability of a definitive trial to test effectiveness of HENRY (local authorities will be ask to nominate four or six centres, depending on availability of eligible centres)

This is a multi-centre, open labelled, two group, prospective, cluster randomised, controlled, feasibility study aiming to recruit 120 parents from 12 children’s centres (Fig. 1).

As the progression criteria indicates, the feasibility study will monitor our ability to recruit centres and will therefore allow a local authority to allocate four rather than six centres in order to answer all objectives of the study. In this situation, trial progression criteria will be highlighted as ‘Amber’. Cluster randomisation has been chosen to reduce contamination as HENRY is a group-based intervention which aims to provide practical guidance and improve parenting skills, intended at enhancing family homes *and* children’s centre environments. In centres allocated to receive HENRY, parent recruitment and registration will be restricted to only those enrolled on a HENRY programme. Centre staff and participants will, of necessity, be aware of treatment allocation, but collection of outcomes will be performed by interviewers from the National Centre for Social Research (NatCen; a social research unit geographically located across the UK) who will be blinded to treatment allocation.

Within each of the two local authorities nominating six centres, three centres will be randomised to HENRY and three will be randomised to the control arm (training to deliver the HENRY programme in these centres will be made available (though not compulsory) at the end of the study). Historically, when HENRY is commissioned within an area, the local authority determines which centres will receive the intervention. In areas where HENRY is provided by external partners (e.g. charities such as Banardos), this is determined by the service provider. To maintain some autonomy for the commissioners, local authorities/service providers will be asked to allow randomisation of half of the centres they propose to the intervention and half to the control arm.

### Unit of randomisation

Following relevant approvals, up to six centres (minimum 4) within each local authority will be randomised to the intervention or control in a 1:1 allocation ratio (HENRY; control) by the Leeds Clinical Trials Research Unit (CTRU) statistician (MC). Minimisation, will be used to ensure the treatment groups are well balanced for size of children’s centre (numbers of permanent centre members of staff not including staff using the centre such as health visitors and nursery workers) (≤eight/>eight members of staff); area level ethnicity (< 80%/≥ 80% White British (Census data)); and area level deprivation (≤ 10%/> 10% ranking within Index of Multiple Deprivation at the Lower Layer Super Output Area).

After randomisation, the CTRU will notify HENRY central office, the local authority lead and the children’s centres of the outcome in order to instigate necessary training arrangements. Notification will be via a secure file transfer system. NatCen will not be notified to maintain blinding of the central NatCen researchers as well as the NatCen interviewers performing the recruitment and data collection.

### Setting

We plan to recruit two local authorities from within any area across England or Wales from which children’s centres will be recruited. Within eligible and interested local authorities, centres will be nominated by the commissioning leads. Following fully signed local authority (and service provider if applicable) and children’s centre agreements, parents will be screened initially by staff within children’s centres. Provided parents’ consent to share contact details, the details of those potentially eligible and interested will be sent to interviewers at NatCen, who will conduct another screening assessment over the phone and book home appointments for those remaining eligible and interested. Eligibility criteria will be confirmed during the home visit, where parents will be provided further opportunity to discuss the study information sheets and asked to provide consent by the NatCen interviewer.

### Eligibility criteria

Note: Study progression criteria requires a minimum of six centres to be recruited within each local authority to meet ‘Green/Go’ criteria. However, in order to fulfil all study objectives, a minimum of four centres will be accepted. In this eventuality, study progression criteria will be labelled as ‘Amber’.

### Inclusion criteria

#### Local authorities (and service providers if applicable)

The inclusion criteria for local authorities (and service providers if applicable) are as follows:

- Local authorities must formally agree to nominate four/six children’s centres and allow randomisation of these centres. This will be evidenced by agreements signed by local authority leads. Where possible, centres should be in geographically separate areas to protect against contamination (judged on a case by case basis). Local authorities using external teams outside of the centre to deliver HENRY programmes (e.g. health visitors) will be eligible, in addition to those wishing to train internal centre staff to deliver programmes (the most common model currently used).

- Local authorities may be completely new to HENRY (having never commissioned, trained or delivered HENRY) or contain four/six centres that are within HENRY ‘naïve’ clusters. For the purposes of this study, HENRY naïve clusters are defined as a group of centres within a cluster (children’s centres that are grouped for management purposes) that do not include any centres that are either (a) currently delivering HENRY, or have delivered HENRY within the past 2 years, or (b) have been trained to deliver HENRY within the past 2 years.

#### Children’s centres

The inclusion criteria for children’s centres are as follows:

- Any type of children’s centre or other early years setting such as a nursery or community venue.
- Children’s centres must aim to run HENRY programmes starting within 4 weeks of training completion and aim to run three courses during the study.
- Children’s centre managers must agree to support participant recruitment within their centres. This will be evidenced by agreements signed by children’s centre managers

#### Parents

The target population for the intervention are parents of preschool children: mothers, fathers and carers (e.g. with children living in stable/long-term foster care).

- Parents must have at least one child aged between 6 months and 5 years. If more than one child in the family fulfils eligibility criteria, the youngest child (by birth timing if twins) will be considered as the reference child (about whom data will be collected).
- Parents must be willing to attend the programme sessions (intervention centres) and willing to provide data in accordance with the data collection protocol. Parents will be provided with full details of the data collection requirements in advance so that they can make informed decisions as to whether to participate.
- Parents must speak English (the intervention and data collection forms are currently only available in English), unless they wish to bring their own interpreter with them (e.g. family member).

### Exclusion criteria

#### Local authorities (and service providers if applicable)

The exclusion criteria for local authorities (and service providers if applicable) are as follows:

- Located in areas with no coverage of NatCen, the organisation responsible for collecting data.

#### Children’s centres

The exclusion criteria for children’s centres are as follows:

- Children’s centres that have delivered HENRY programmes within the last 2 years or where staff have received HENRY training within the last 2 years.

#### Parents

The exclusion criteria for parents are as follows:

- Parents with severe learning difficulties that preclude them taking part in group sessions (in which they need to be able to read and write) or results in insufficient capacity to consent. This will be judged on a case by case basis with consultation with the HENRY team where appropriate.
- Parents whose reference child is tube fed (PEG or nasogastric) or with other known clinical conditions likely to affect growth over the period of the trial (e.g. cancer, coeliac disease or renal or cardiac problems). A detailed list of excluded conditions will be provided at screening, with any uncertainties resolved via clinical input from the HENRY team.
- Parents who have attended a HENRY group for a previous child.

### Recruitment and consent

**Fig. 2:**
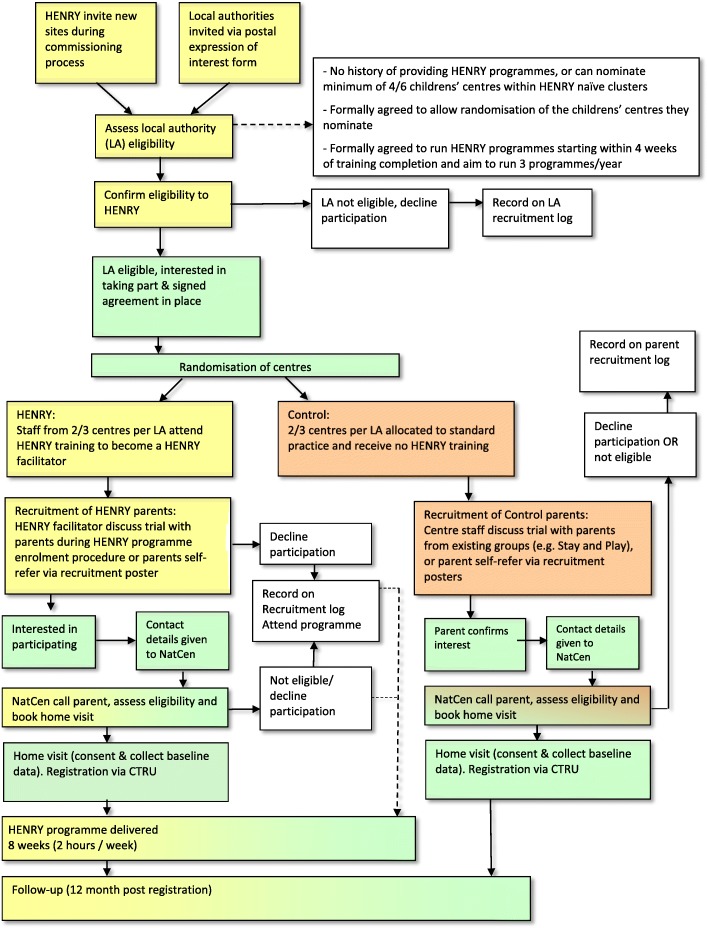
Recruitment pathway: identification and consent

Figure 2 details the stages of recruitment.

#### Local authorities

Recruitment of local authorities will be done in two ways. Firstly, authorities that make enquires to the HENRY central office will be invited to take part during standard commissioning conversations. Second, local authorities that have (a) previously enquired, but not yet commissioned HENRY, over the previous 12 months or (b) currently commission HENRY will be invited to take part via postage invitation mailed from the CTRU. In the latter, only authorities with centres within HENRY naive clusters (i.e. not running HENRY programmes) will be eligible (see the ‘Eligibility criteria’ section). A designated member of staff at the HENRY central office will be responsible for liaising with commissioners (new and existing). They will be provided with expression of interest forms, including an eligibility checklist to use during these conversations. This will allow commissioners to learn about the study. All expression of interest forms and eligibility checklists will be sent to CTRU and recorded on a local authority recruitment log. In order to gain an understanding of the potential number of local authorities that would be interested in participating (as we are only recruiting two), expression of interest forms will also be mailed from the CTRU to all local authorities assumed to meet eligibility criteria (judged by HENRY records of training).

After being informed of the study, local authorities will be given up to 4 weeks to decide to participate (allowing time for necessary meetings/discussions to occur). Areas expressing an interest in participating will be asked to (1) nominate a minimum of four children’s centres and (2) sign an agreement before progressing to the study. In areas where children’s centres are tendered to service providers, study details and agreements will be sent to these service providers in addition to the commissioning lead. Both the commissioner and the service provider need to agree to participate in order to take part. Areas declining participation or not responding will continue with their standard practice and/or HENRY commissioning processes if applicable. Basic demographic information and reasons for declining participation (where appropriate) will be recorded on a local authority recruitment log. Consent to participate in the study will be assumed via signing of the agreement.

#### Children’s centres

Children’s centres within each local authority will be nominated to take part in the study by the local authority/service provider commissioning lead. The choice of centres to deliver HENRY programmes is traditionally based on a number of factors, including perceived level of need and/or deprivation. This process will continue, although local authorities will nominate approximately twice the number of centres that they wish to commission HENRY, so that half can be randomised to receive HENRY and half can be randomised to control. Children’s centres will receive £200 per centre to cover administration costs associated with supporting parent recruitment, which will be paid at the start of the study on confirmation of approval that the centre will take part. Researchers at CTRU will send a children’s centre agreement to the children’s centre manager to explain that their local authority has decided to commission HENRY and has agreed to be part of the study. Commissioning leads will be encouraged to discuss this with centre managers in advance so that they are already familiar with the details. Information on the manager’s delegation of duties will be provided within the agreement and they will be informed that they will be randomised to receive HENRY or be in the control arm; both of which will require support to recruit parents. Consent at this level will be evidenced by children centre manager signature of the agreement.

#### Parents

Parents attending participating children’s centres will be approached by children’s centre staff, who will introduce the study.

In centres randomised to HENRY, parents will be recruited when they enrol to attend the HENRY programme. This procedure has been used successfully in the past and indicates that at least four parents within each programme will be eligible and will agree to take part in the study [18]. In centres that have been randomised to the control arm, parents will be recruited from other programmes that are running within the Centres such as ‘Stay and Play’. In both HENRY and control centres, staff will introduce the trial to parents and request that all parents approached complete a parent screening form collecting anonymised data on age range, ethnicity and basic eligibility criteria. Parents will indicate on the screening forms if they are interested in taking part in the study and, if interested, asked to provide consent to give their contact details to a NatCen interviewer for confirmation of eligibility. Parents will be informed that providing data collected for screening is not compulsory and that all data provided will remain anonymous.

Upon receipt of the parent screening forms (including their consent to be contacted), a local NatCen interviewer will call parents to provide further details of the study and confirm eligibility. NatCen are an independent social research agency (http://natcen.ac.uk) that has no role in delivery of the intervention. Informed written consent and recruitment will occur in parent’s homes and will be undertaken by interviewers from NatCen. Parents will be provided participant information sheets disclosing the full details of the study and their requirements including the feasibility nature of the study and its impact on future research. Prior to providing consent, they will be given time to discuss the study and ask any questions. All parents will have the right to refuse consent without giving reasons and will remain free to withdraw from the study at any time without giving reasons.

Parents who are ineligible or decline will continue to attend HENRY or equivalent sessions. Their anonymised details (age range, ethnicity and eligibility data) will be recorded in a recruitment log. In both HENRY and control centres, parents will have the opportunity to self-refer into the study via recruitment posters displayed in the children’s centres. Where a parent has learnt about the study via a recruitment poster and wishes to self-refer, they will contact a CTRU researcher directly using the contact information provided within the poster, who will conduct the initial screening and consent for researcher contact prior to notifying the NatCen interviewer.

Across all methods of recruitment, parents will be offered feedback on child’s growth and habits at the end of the study and will receive a £10 shopping voucher at baseline and follow-up (£20 total) provided by the NatCen interviewer during visits.

### Blinding

The NatCen interviewer will be blinded to whether the participant was recruited via a children’s centre receiving HENRY or acting as a control. Parents will be asked not to divulge this information to NatCen by centre staff during screening. This will be repeated in the information sheets and verbally by the NatCen interviewers at the start of all telephone calls and in-person assessments. Where the NatCen interviewer does become aware of participant allocation, an un-blinding form will be completed. As the participant approaches 12 months post registration, they will be contacted again by the NatCen interviewer to arrange a second visit where 12-month follow-up data will be collected. Attempts will be made to use different interviewers at follow-up if the interviewer who conducted the baseline assessment is aware of participant allocation.

Feedback from our parent advisory group suggests that some parents/carers may prefer a visit at their centre rather than in their home. This will be possible if requested; however, as HENRY programmes will be advertised within centres, it may lead to un-blinding of the NatCen interviewer. Thus, location of all visits and assessments will be recorded and un-blinding will be assessed.

### Intervention

#### HENRY

HENRY (Health, Exercise, Nutrition for the Really Young) is an 8-week programme delivered in children’s centres, aiming to provide parents with skills and knowledge to support healthy lifestyles in preschool children and their families. Two stages of training for children centres staff include [21] the following:

- *Stage 1: Centre level training:* 2-day training to equip staff with knowledge and skills to promote and provide healthy nutrition within early years settings and support parents to provide healthy family lifestyles and nutrition for their families. The theoretical underpinning combines proven models of behaviour change including the Family Partnership Model, motivational interviewing and solution-focused support.
- *Stage 2: Practitioner level training to deliver the ‘HENRY’ programme to families:* After completing stage 1, practitioners can be trained to deliver the 8-week HENRY programme, which aims to build parents’ skills, knowledge and confidence to change old habits and provide healthier nutrition for their young children. Earlier evidence suggests that it is enjoyed by parents [22] and national evaluation data provides some indication of success [18]. Similar to the centre level training, the approach is strength based and solution focused. Programme content includes sessions on lifestyle and eating habits (e.g. family meals), balancing healthy meals and snacks, and child appropriate portion sizes with the aim to support families to optimise parenting skills, eating patterns and behaviour, healthy eating, physical activity and emotional well-being.

### Control

In centres not allocated to receive HENRY training (control arm), standard practice will continue until the end of the study (after all follow-up data have been gathered) when children’s centres will be offered free training to allow them to deliver HENRY programmes under the terms of their HENRY licence. Standard practice will entail families receiving the standard level of support provided by children’s centres. Services provided by children’s centres are aimed at supporting young children and their families, with a particular focus on the most disadvantaged families, in order to reduce inequalities in child development and school readiness [23]. These services are varied but often include health services (e.g. access to health visiting teams, breastfeeding support), parenting advice, healthy eating advice and access to specialist services (e.g. speech therapy, money management). The availability of services, deemed similar to HENRY (e.g. healthy eating, parenting) and those attended by parents during the study will be recorded via baseline and follow-up questionnaire. Due to the classification of HENRY naïve clusters as those where centres have not delivered a HENRY programme or received HENRY training within the last 2 years, it is possible that participating children’s centres could have delivered HENRY programmes in the past. Therefore, centres within control areas could potentially be consistent with the HENRY approach. This potential dilution effect will be explored via a baseline and follow-up environmental questionnaire (gathering data on centre policy and practices) and the contamination element of the process evaluation which will shed light on where contamination might exists between HENRY participants and non-HENRY participants attending centres that are consistent with HENRY principles and what impact this contamination may have.

### Measurement

The feasibility objectives will be determined through analysis of process data, routinely collected data from HENRY central office and the quality of outcome data. Process evaluation will determine fidelity, dose and quality of HENRY programme implementation using a combination of quantitative and quality data. Quantitative process data will be obtained through HENRY quality assurance processes which include HENRY central office assessment of whether key performance indicators have been met by facilitators during delivery of the programmes. HENRY facilitators are also required to complete an end of programme review reflecting on how the session was delivered and received. HENRY central office will also provide confirmation of facilitator training certification and participant attendance records for parents allocated to attend a HENRY programme. Quality assurance measures will be discussed and recorded at monthly meetings between HENRY and CTRU. Participant attendance data will be transferred to CTRU by secure transfer encrypted system using a data specification document provided by CTRU. Data transfer agreements (including details on the sender, recipient, content of transfer and any data-processing limitations) will be set up between HENRY and the CTRU in advance. These data will be combined with other qualitative data as part of a wider process evaluation by a member of the trial team (WB), gathered at multiple time points throughout the study in line with each period of recruitment (see the ‘Process evaluation’ section). Contamination will be assessed using a combination of quantitative (e.g. data on facilitators working in multiple centres) and qualitative (e.g. parent focus groups) measures by mapping intervention components against their potential to impact on outcomes.

Parental self-report questionnaires at baseline and 12 months will be administered by NatCen interviewers within family homes, or at the children’s centre if preferred by the parent. Paper-based case report forms will be completed using an interview administration process by trained, blinded researchers from the NatCen workforce. Completed case report forms will be mailed by NatCen interviewers to the CTRU at monthly intervals. These will not contain identifiable information (unique identifiers only).

### Feasibility outcomes

The feasibility outcomes were as follows:

- Recruitment rate, including number of local authorities screened for eligibility, reason for ineligibility and number (and reason) for declining; number agreeing to take part (including number of nominated centres); number of centres agreeing to take part (and reason for those declining); number of parents approached; number (and proportion) agreeing to researcher contact; number (and proportion) of eligible parents consenting; number of parents self-referring; and reasons for parent ineligibility and non-consent.
- Training and quality assurance, including length of time taken to train and certify staff to deliver HENRY programme; number of staff attending training; number of staff certified to delivery HENRY; changes in knowledge and practice before and after training (from pre and post training facilitator questionnaire); and programme delivery quality assurance (collected routinely by HENRY).
- Contamination identification and risk, ascertained through determining the number of parents registered to control centres attending centres running HENRY; HENRY-trained facilitators working in or visiting control centres and proportion of their time spent working in other centres; control and intervention centres merging with other centres; other schemes addressing lifestyle change attended by parents during the study; parents registered to control centres who have had contact with parents attending centres running HENRY. We will also assess the frequency of contact between parents in HENRY and control centres. Risk will be estimated by triangulating identified sources of contamination with outcome data and qualitative assessment of perceived contamination.
- Acceptability of data collection protocol, assessed from the number, proportion and timing of parent withdrawals from HENRY programme (intervention centres only) and follow-up data collection (both parents registered to HENRY and control centres) out of those registered and reasons for withdrawal; number (and proportion) of registered parents lost to follow-up; number (and proportion) of parents with self-reported questionnaire and height/weight data; and missing item level data on questionnaires. Feedback will be gathered from parents, centre staff and commissioning leads via interviews and focus groups.
- Determination of sample size for definitive trial estimated using feasibility data: variability of gender-adjusted body mass index (BMI) in both arms; difference between arms and 95% confidence intervals; and estimation of clustering effect (ICC) and cluster size.
- Intervention compliance and implementation, estimated from routinely gathered data on timing of delivery of first HENRY programme (plus reasons if delivery occurs later than 4 weeks after training); number of HENRY courses delivered per centre; and attendance rates at HENRY programmes and reasons for absence.

### Intervention impact measures

Data from the following measures will be collected at the baseline and follow-up to support the feasibility objectives. Provided they are deemed feasible and acceptable, they will be considered for inclusion in a future definitive trial.

#### Primary outcome

The primary outcome was reference child BMI z-score (age- and gender-adjusted height(m)/weight(kg)^2^) measured by NatCen interviewers.

#### Secondary outcomes

Secondary outcomes were as follows:

- Primary caregiver BMI (measured height(m)/weight(kg)^2^) and waist circumference (cm).
- Family eating/activities using the validated Golan Family Eating and Activity Habits Questionnaire (21 items) [24] assessing activities such as leisure time activities, eating habits and style, hunger and satiety cues, exposure to and availability of problematic foods and stimulus control, and frequency of parent and child eating meals and snacks together.
- Parenting self-efficacy, gathered via the Dumka Parenting Self Agency Measure (5 items) [25]. This questionnaire measures parents’ overall confidence in their ability to act successfully in their parental role.
- Feeding, collected via the Baughcum pre-schooler feeding questionnaire (37 items) [26]), used to measure maternal feeding practices, child eating behaviours and maternal beliefs.
- Dental health via a bespoke questionnaire based on the Dental Health Survey of Children and Young People by the University of Leeds, School of Dentistry, to measure the potential wider impact of HENRY and a child’s dental health (Dental Questionnaire). This contains 6 questions related to tooth brushing and dental attendance and whether the child has received general anaesthetic treatment.

### Process evaluation

As this study is aimed at determining the feasibility of undertaking a definitive trial to evaluate the clinical effectiveness of the HENRY programme, the focus of the process evaluation will be to measure contamination, acceptability and implementation. The process evaluation will follow guidance from the Medical Research Council (2015) and measure relevant constructs proposed by Baranowski and Stables [27]. This evaluation will also facilitate accurate reporting requirements of the TiDieR checklist [28]. The objectives of the process evaluation are the following:

- To explore the extent of contamination between intervention and control arms by monitoring staff and facilitator overlap between centres via staff movement questionnaire and holding interviews with managers, facilitators and staff to assess how adoption (or previous adoption) of the HENRY approach influences individual and centre-level practice.
- To explore contamination and associated impact on outcomes between parents through social networks by holding focus groups with parents from intervention and control arms.
- To determine the extent to which all programme sessions are delivered as planned, including dose and content delivered, quality, schedule and duration via ongoing report from HENRY central office who routinely measure the implementation and quality assurance of programmes.
- To investigate barriers to maintaining delivery of the programmes over the trial period at the local authority and children’s centre level via interviews with managers and local authority commissioners.
- To determine the acceptability of recruitment processes and data collection methods by holding interviews with commissioners, managers, staff and parent focus groups). NatCen fieldworkers will also provide feedback on the acceptability of baseline and follow-up visits via formal debriefing sessions.

Qualitative data collection will occur at multiple time points throughout the study. Local authority commissioner interviews will be held approximately 3–6 months from randomisation of children’s centres (to allow time for centre set-up) to discuss their views on barriers and levers to taking part in the research. Manager interviews will take place 12 months after centres opening to recruitment to enable them to draw on their experience of the study as a whole to discuss barriers to implementation and potential sources and impact of contamination. Interviews with children’s centre staff and HENRY facilitators, and focus groups with parents will be held periodically throughout the study, coinciding with the end of each HENRY programme, where acceptability and contamination will be explored.

All interviews and focus groups will be transcribed by members of the research team and imported in to NVivo data analysis software [29] to assist with coding and management. Qualitative data analysis will be guided by a deductive organising framework devised from the process evaluation research objectives (contamination, implementation and acceptability) and interview and focus group topic guides. Inductive thematic analysis will then be applied to identify codes and sub-codes, and potential relationships between these codes [30]. The primary coding of qualitative data will be undertaken by WB followed by second coding from another team member. Ongoing analysis will discussed with the wider research team and consensus reached for the content of themes and their influence on the subsequent design of a definitive trial.

### Health economics

The Health Economics component of a future definitive trial will aim to assess the within-trial cost-effectiveness of HENRY to reduce preschool obesity. For the feasibility study, a full cost-effectiveness analysis will not be conducted, but we will check the completeness and the ability to obtain quality of life (EQ-5D) [31] and health care resource use data for the child’s health and the parent within the NHS (health services, hospital, social services) as well as time off work in relation with HENRY. The feasibility study will therefore be an opportunity to identify the most appropriate way to collect the data needed to assess the cost-effectiveness in a definitive trial.

A study-specific health resource use questionnaire will be used to collect information on primary and secondary health care utilisation for both the parents and the child at follow-up. We will collect out of pocket expenses such as parents’ expenses in relation with travel for the intervention and other private expenses (e.g. extra food expenses, extra activities) to improve child’s diet. We will also collect parents’ lost productivity (time off work) in relation to child’s health and attendance to the programme.

Wherever possible, unit costs for resources will be obtained from national sources such as the British National Formulary [32] and the PSSRU Costs of Health and Social Care [33]. NHS and social service resource use will be identified through direct observation of the treatment provided within the feasibility study and through the structured questionnaire for collection of all other service use.

### Statistical analysis

Recommendations for feasibility studies suggest that at least 30 participants per group will allow sufficient precision when estimating study summary measures [34]. We plan to recruit two local authorities and 120 parents to give confidence that a phase III multi-centre trial can be successfully conducted [34, 35].

As this is a feasibility study, the analysis will focus on descriptive statistics and confidence interval (CI) estimation rather than formal hypothesis testing (i.e. no formal evaluation of the study interventions will be conducted as part of this feasibility study). The trial is not powered to provide a precise estimate of the level of clustering relating to group effects, but it will allow an investigation of this effect, which will inform the sample size estimation for the definitive trial. No formal analyses are planned until after the trial is closed to recruitment and follow-up and the required number of local authorities/centres have been randomised and the required number of parents have been registered. Final analysis will be carried out when all available outcome data has been collected.

The feasibility and success of the recruitment strategy will be evaluated by summarising the screening, eligibility, consent and randomisation/registration processes, including the numbers of local authorities/centres/parents involved during each stage. Reasons for non-participation in the study will be summarised. The total number of parents registered will be summarised, overall, by month/programme (as appropriate) by centre and local authority. The length of time taken to train and certify staff to deliver the HENRY programme will be detailed by HENRY staff. The number of staff attending training/certified will be summarised overall and by centre. Facilitator response to training will be captured as part of the process evaluation.

Participant retention during follow-up, including the number of centres/parents withdrawing from the study and the timing of and reasons for the withdrawal will be presented overall, by arm and time-point. We will also report losses to follow-up over time. We will also report the difference and its confidence intervals (CI) for follow-up rates between the intervention and control groups (to identify large differences between the arms). Levels of missing self-reported outcome data, both at the individual item level and for entire outcome measures will be reported overall, by time-point and by treatment arm.

Centre environment, family eating/activities, parenting self-agency, pre-schooler feeding and dental health at baseline and at 12 months post registration will be summarised overall, by arm and by time-point and where appropriate by centre (centre environment). 95% CI will be constructed for the differences in these outcomes between control and intervention groups. Mean activity per day will be summarised overall, by arm and by time-point.

In order to inform the sample size calculation for the definitive trial, we will calculate the gender-adjusted BMI, overall and per arm together with corresponding 95% CI. We will also compare the variability of gender-adjusted BMI between arms. As variation surrounding the intra-class correlation coefficient (ICC) estimate is likely to be large, we will estimate the ICC after controlling for covariates and constructing a CI [34, 36] to obtain a range of plausible estimates from which a variety of sample sizes can be calculated. A conservative approach would be to use the 80% upper one-sided confidence limit of the ICC estimate [34] in the sample size calculations.

To assess compliance with and fidelity of delivery of HENRY, the timing of delivery of the first HENRY programme will be summarised overall and by centre. Reasons for delivery post 4 weeks after receiving training will be presented by centre. In addition, the number of HENRY programmes delivered per centre, attendance rates and reasons for absence will be presented.

### Data monitoring and confidentiality

Missing local authority and children’s centre demographic and screening data will be chased until it is received, confirmed as not available or if the analysis by CTRU has started. Parent data will be monitored for quality and completeness by the NatCen research team prior to its transfer to the CTRU. Data provided to the CTRU will be monitored for quality and completeness by the CTRU. Monthly data query reports will be generated by CTRU and forwarded to the NatCen research team.

All data provided will be stored, handled and processed in accordance with the principles of the 1998 Data Protection Act and ISO 27001, and specific strategies will be used to maintain anonymity and confidentiality. Operationally, this will include the following:

- Local authority and parent level screening data (containing data on local authorities/centres/individuals that decline).
- Where documentation is required that includes identifiable information from sites, e.g. postcode, data will be posted to the CTRU and stored securely in a separate location to study data.
- Participant contact details will be collected by children’s centres during screening and posted to NatCen to facilitate study registration. These data will be posted to NatCen in batches via secure tracked mail.
- Participant contact details will be transferred to CTRU so trial reminders can be sent and a selection of participants can be invited to take part in the focus group. This data will be transferred to CTRU via the secure data transfer system and stored securely at CTRU in a separate location to study data.
- All study data collected from participants will be transferred from NatCen to CTRU via post and will be coded with a unique study ID and two identifiers (the participant’s initials and date of birth).
- If a local authorities/centres or parents withdraw consent from further collection of data, the existing data that they have provided up to the date of withdrawal will remain on file and will be included in the final study analysis.

### Trial oversight

The Trial Steering Committee will provide oversight for the study. In particular, they are responsible for monitoring the study progress, adherence to protocol, participant safety and consideration of new information. It includes an independent chair and three other independent members, including an experienced trial statistician. The chief investigator and other members of the internal project team attend all FSC meetings and present and report progress. The FSC operates in line with the CTRU’s Committee terms of reference (ToR) as amended and agreed by FSC members at their first meeting.

A Study Management Group (chaired by Hartley), comprising the chief investigator, CTRU team (Burton, Collinson, Hartley) and health economist (Tubeuf) will be assigned responsibility for the set-up, ongoing management and promotion of the trial. This includes (i) protocol completion, (ii) data collection requirements, (iii) obtaining ethical approval, (iv) completing cost estimates and project initiation, (vi) facilitating the FSC, (vii) monitoring of site conduct and (vii) interpretation of results and contribution to publications. The SMG will operate in line with the agreed ToR.

For a trial of this nature and duration, a separate Data Monitoring and Ethics Committee is not required. Rather, the Steering Committee will adopt a safety monitoring role, with the constitution of a subcommittee to review safety issues where this becomes necessary.

The sponsor (University of Leeds) has the right to conduct source verification although this is not anticipated given safety and efficacy are not endpoints of this study. The rights for the data belong to the Study Sponsor, and no processing, including further data transfer in whole or in part to a 3rd party, is permitted other than as stated in the data transfer agreements.

To ensure responsibility and accountability for the overall quality of care received by participants during the study period, clinical governance issues pertaining to all aspects of routine management will be brought to the attention of the Trial Steering Committee and, where applicable, to individual local authorities and/or children’s centres. NatCen interviewers will follow standard NatCen safeguarding procedures. If a disclosure issue is raised by an interviewer during a home visit, details will be sent via email to the Director of Field to review, and, if necessary, the issue will be referred to the NatCen disclosure board.

### Trial organisation and administration

The trial is sponsored by the University of Leeds and co-ordinated by the CTRU (Clinical Trials Unit, University of Leeds, LS2 9JT). The management group consists of the chief investigator and the study management group. Protocol amendments will be handled in accordance with relevant Health Research Authority (HRA) guidance and CTRU standard operating procedures. Amendments required to the protocol and ethically approved documents will be identified by the trial researcher and the chief investigator and submitted to the School of Medicine Research Committee for ethical opinion prior to implementation. A list of amendments will be included in the final report.

A parent advisory group (consisting seven parents of preschool children) has been convened to support all aspects of the trial. The parent advisory group meet locally twice a year to provide feedback on aspects such as study design (e.g. consideration of control group), parent recruitment (e.g. strategies to recruit and recommendations for incentives) and data collection (highlighting sensitive items requiring more consideration). Moving forward, they will also help interpret the data and with engagement and dissemination activities. Feedback from the parent advisory group meetings is reported back to the steering committee via the research team and via a member of the parent advisory group who also sits on the Trial Steering Committee.

### Publication policy

The success of the research depends upon the collaboration of all partners. For this reason, credit for the main results will be given to all those who have collaborated in the research, through authorship and by contribution. Uniform requirements for authorship for manuscripts submitted to journals will guide authorship decisions (www.icmje.org). These state that authorship credit should be based only on substantial contribution to the following:

- Conception and design, or acquisition of data, or analysis and interpretation of data
- Drafting the article or revising it critically for important intellectual content
- Final approval of the version to be published
- And that all these conditions must be met.

Other acknowledgements to the steering committee, the parent advisory group, members of the HENRY team, NatCen, participants and others will be agreed and included as appropriate.

The first author is responsible for the submission of the publication and must keep the HENRY central office team and all authors informed of the abstract’s or manuscript’s status. The steering committee will be kept informed of rejections and publications as these occur. On publication, the first author should send copies of the abstract and manuscript to the steering committee, the SMG, the Sponsor and to all other co-authors and ensure communication with the funder as per the contractual agreement.

## Discussion

Childhood obesity impacts physiological and psychological health that tracks into adulthood, increasing risk of morbidity and mortality [37, 38]. It incurs significant costs on the UK economy, with an expected sevenfold increase in related NHS costs by 2020, and forecasted £2 billion annual spend by 2030 [39]. Tackling obesity is a key NHS public health priority, and programmes to treat and prevent it are commonly commissioned in local authorities. Evidence of such programmes from randomised controlled trials is lacking. It is acknowledged that many local authorities commission a range of programmes, despite their lack evidence base. The urge to favour action over evidence is understandable given current rates of childhood obesity. However, we argue that demonstration of effectiveness and cost-effectiveness is fundamental to support the prioritisation of tax payer’s spending. The HENRY programme is an intervention with preliminary evidence suggesting high levels of parent acceptability and trends in increasing positive healthy weight behaviours in those attending [18, 22]. Over 10,000 families have already attended the HENRY programme, funded through public money; it is therefore important to establish its effectiveness.

The proposed feasibility study will support decision making in a future definitive trial. If any of the progression criteria are not met, future assessment of the effectiveness of the HENRY programme will be negotiated. Other findings such as the identified risk and impact of contamination will support a potential re-design of a trial, for example, to consider the ability to conduct individual level randomisation in existing HENRY areas to increase our recruitment pool. However, further feasibility testing may be needed prior to the conduct of a definitive trial in this eventuality. During our design phase, we undertook consultation with stakeholders, including local authorities, parents, our Trial Steering Committee, HENRY central office and methodologists within the CTRU. There was initial concern that the local authority commissioners would not agree to have centres randomised to HENRY or not, particularly as such programmes are often commissioned to support disadvantaged families. An alternative stepped wedge design was proposed so that, by the end of the trial, all centres would be randomised to HENRY. While deemed to be acceptable, issues with contamination (e.g. control parents attending the same centre as active parents), the requirement to host multiple training sessions after each randomisation and the impact of calendar time and facilitator learning curve on outcomes meant that a stepped wedge design was not pursued further. Importantly, our discussions with commissioners revealed that they were happy with both the parallel and stepped wedge designs.

The progression of this feasibility study to a definitive trial of effectiveness will provide an evidence base of an approach to prevent childhood obesity, which is attractive to all stakeholders, including parents. If deemed effective, HENRY offers extensive benefit to health, including reduced preschool obesity, improved nutrition/eating behaviours in children, healthier family lifestyles and enhanced parenting skills, reducing related morbidity and mortality. Proposed research therefore has an expressed need, not just for obesity prevention, but also to evaluate the return on investment in terms of effectiveness and cost-effectiveness of a programme that has already incurred much spending.

## Abbreviations

BMI: Body mass index (height(m)/weight(kg)^2^)
CTRU: Clinical Trials Research Unit
HENRY: Health Exercise Nutrition for the Really Young
ICC: Intra-class correlation coefficient
NatCen: National Centre for Social Research
TSC: Trial Steering Committee
